# Supplementary material for: Widespread impact of immunoglobulin V gene allelic polymorphisms on antibody reactivity
Source: bioRxiv. 2023 Jun 7:2023.06.06.543969. Preprint. [Version 1] doi: 10.1101/2023.06.06.543969 (PMC10274783; doi:10.1101/2023.06.06.543969)
Supplement: Supplement 1 [file media-1.pdf]

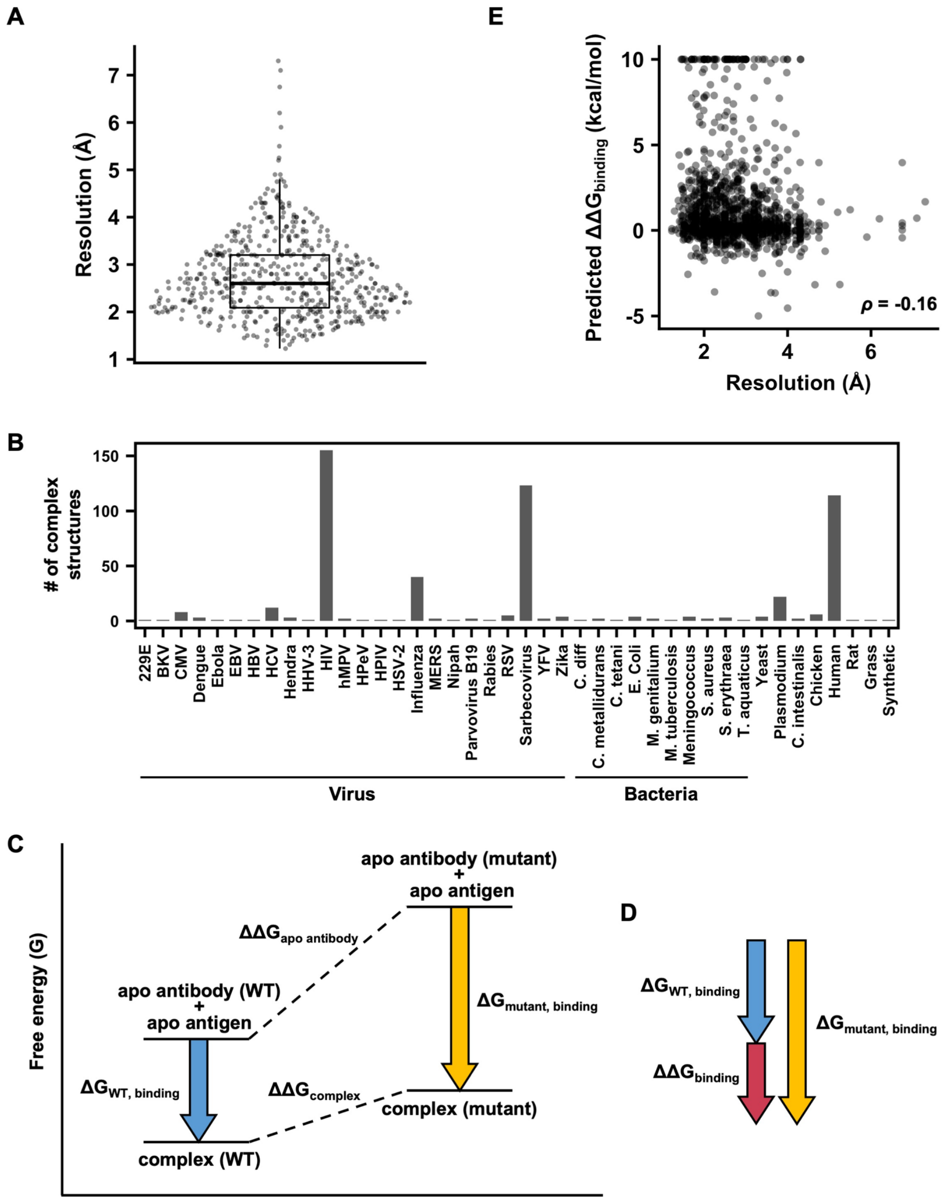

Figure S1. Overview of the dataset and ΔΔG calculation. (A) Resolutions (in Å) of the 544 antibody-antigen complex structures are shown. **(B)** Paratope residues with allelic polymorphisms were identified in 544 antibody-antigen complex structures. The species of the antigen among these 544 antibody-antigen complex structures are plotted, with the occurrence frequency shown on the y-axis. **(C)** A free energy diagram of binding between antibody and antigen is shown. Upon mutation of the antibody, the free energy (G) of both the complex and the apo antibody may change, which can be quantified as $\Delta\Delta G_{complex}$ and $\Delta\Delta G_{apo\ antibody}$, respectively. Blue arrow

indicates the ΔG of binding of the wild-type (WT) antibody ($\Delta G_{WT,\ binding}$), whereas the yellow arrow indicates the ΔG of binding of the mutant antibody ($\Delta G_{mutant,\ binding}$). **(D)** The difference between $\Delta G_{WT,\ binding}$ and $\Delta G_{mutant,\ binding}$ is represented by $\Delta\Delta G_{binding}$, which can be quantified as $\Delta\Delta G_{complex} - \Delta\Delta G_{apo\ antibody}$. That is, $\Delta\Delta G_{binding} = \Delta G_{mutant,\ binding} - \Delta G_{WT,\ binding} = \Delta\Delta G_{complex} - \Delta\Delta G_{apo\ antibody}$. In this example here, the mutation strengthens the binding since it destabilizes the apo antibody to a greater extent than the complex. **(E)** The relationship between resolution and $\Delta\Delta G_{binding}$ of the 1,150 paratope allelic mutations is shown. The Spearman's rank correlation coefficient (*ρ*) is indicated. Mutations with predicted $\Delta\Delta G > 10$ kcal/mol are shown as 10 kcal/mol. Mutations with predicted $\Delta\Delta G < -5$ kcal/mol are shown as -5 kcal/mol. (**A and E**) One data point represents one paratope allelic mutation.

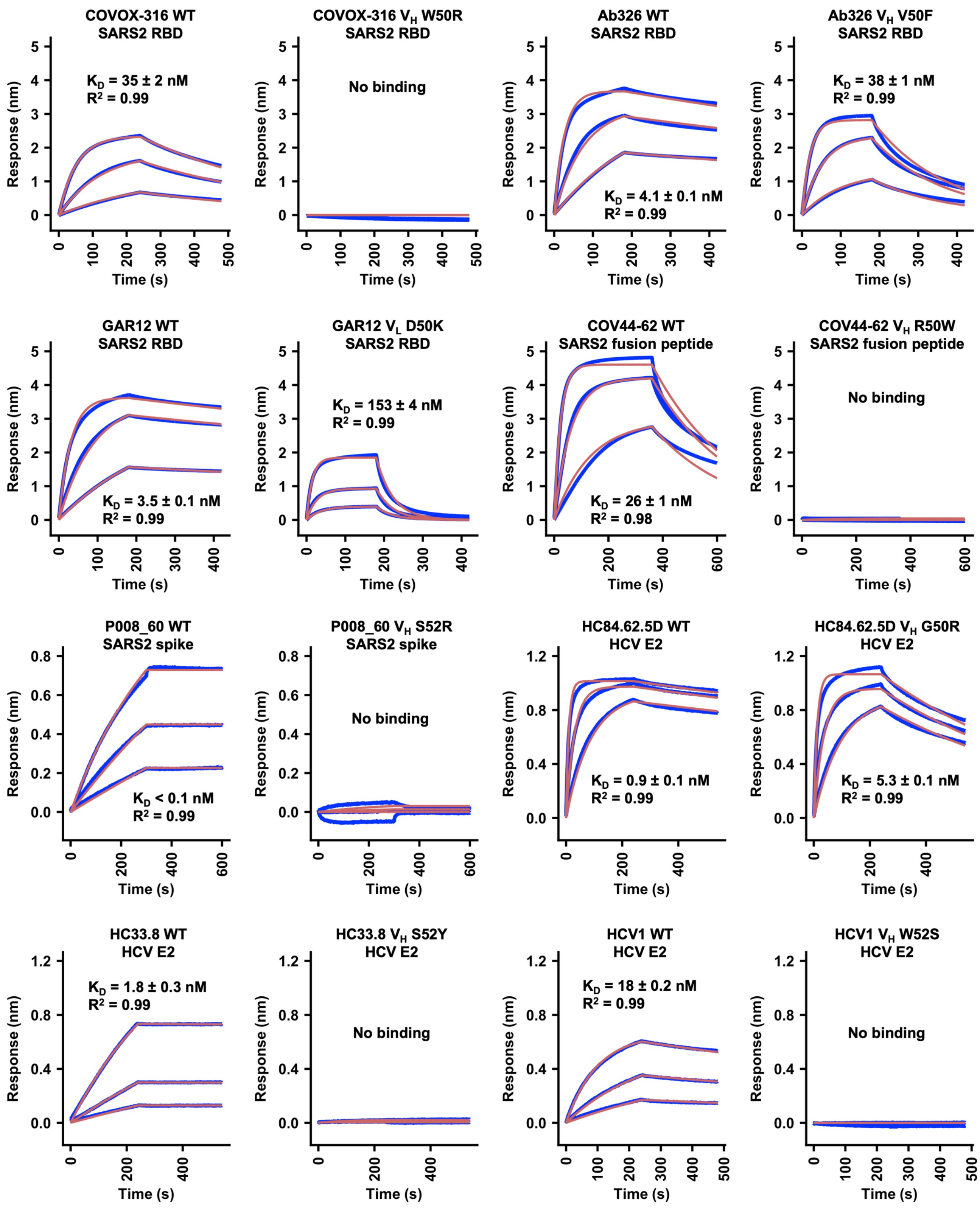

COVOX-316 WT
SARS2 RBD
K_D = 35 ± 2 nM
R^2 = 0.99
COVOX-316 V_H W50R
SARS2 RBD
No binding
Ab326 WT
SARS2 RBD
K_D = 4.1 ± 0.1 nM
R^2 = 0.99
Ab326 V_H V50F
SARS2 RBD
K_D = 38 ± 1 nM
R^2 = 0.99
GAR12 WT
SARS2 RBD
K_D = 3.5 ± 0.1 nM
R^2 = 0.99
GAR12 V_L D50K
SARS2 RBD
K_D = 153 ± 4 nM
R^2 = 0.99
COV44-62 WT
SARS2 fusion peptide
K_D = 26 ± 1 nM
R^2 = 0.98
COV44-62 V_H R50W
SARS2 fusion peptide
No binding
P008_60 WT
SARS2 spike
K_D < 0.1 nM
R^2 = 0.99
P008_60 V_H S52R
SARS2 spike
No binding
HC84.62.5D WT
HCV E2
K_D = 0.9 ± 0.1 nM
R^2 = 0.99
HC84.62.5D V_H G50R
HCV E2
K_D = 5.3 ± 0.1 nM
R^2 = 0.99
HC33.8 WT
HCV E2
K_D = 1.8 ± 0.3 nM
R^2 = 0.99
HC33.8 V_H S52Y
HCV E2
No binding
HCV1 WT
HCV E2
K_D = 18 ± 0.2 nM
R^2 = 0.99
HCV1 V_H W52S
HCV E2
No binding
Response (nm)
Time (s)

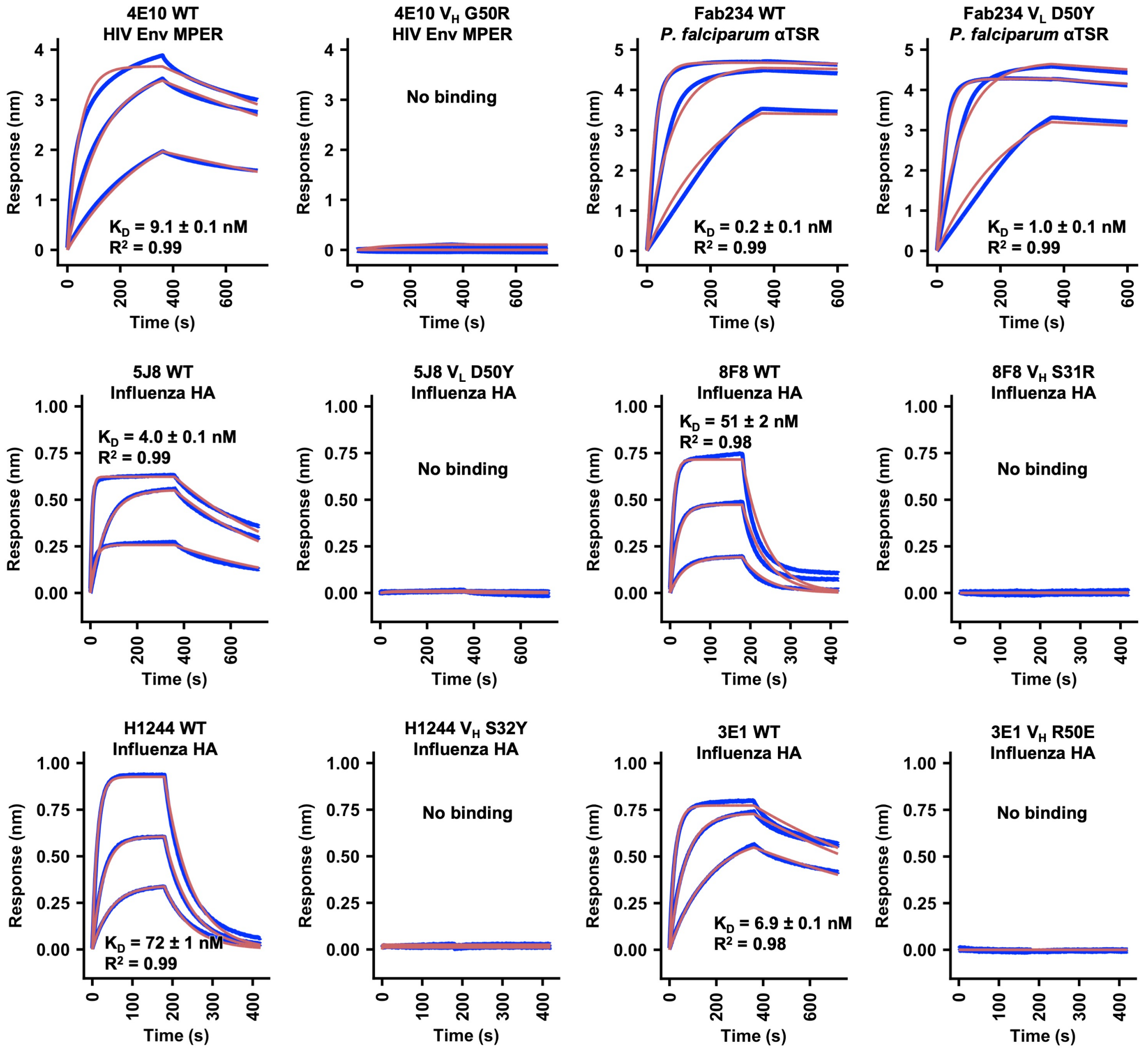

Figure S2. Sensorgrams for binding of Fabs to recombinantly expressed antigens. Binding kinetics of different Fabs against their corresponding recombinantly expressed antigens were measured by biolayer interferometry (BLI). Y-axis represents the response. Blue lines represent the response curve and red lines represent a 1:1 binding model. Binding kinetics were measured for three Fab or antigen concentrations. Dissociation constant (K_D) and the goodness of model fitting (R^2) are indicated.

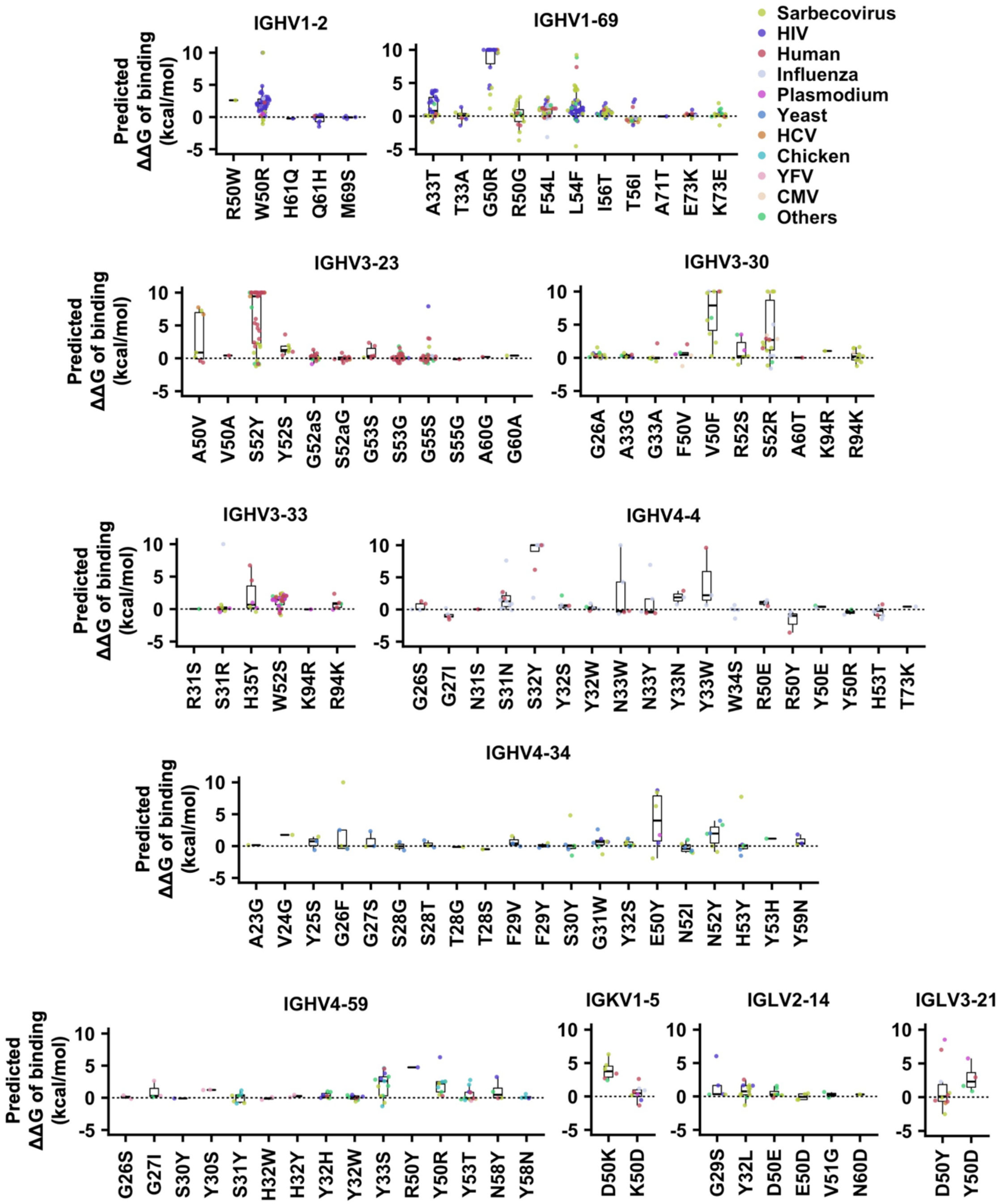

Figure S3. Distributions of predicted $\Delta\Delta G_{binding}$ of paratope allelic mutations. The distributions of predicted $\Delta\Delta G_{binding}$ of paratope allelic mutations in antibodies encoded by the indicated IGV genes are shown. Paratope allelic mutations are categorized by their identities and colored by the antigens. One data point represents one paratope allelic mutation. Mutations with predicted ΔΔG > 10 kcal/mol are shown as 10 kcal/mol. Mutations with predicted ΔΔG < -5 kcal/mol are shown as -5 kcal/mol.

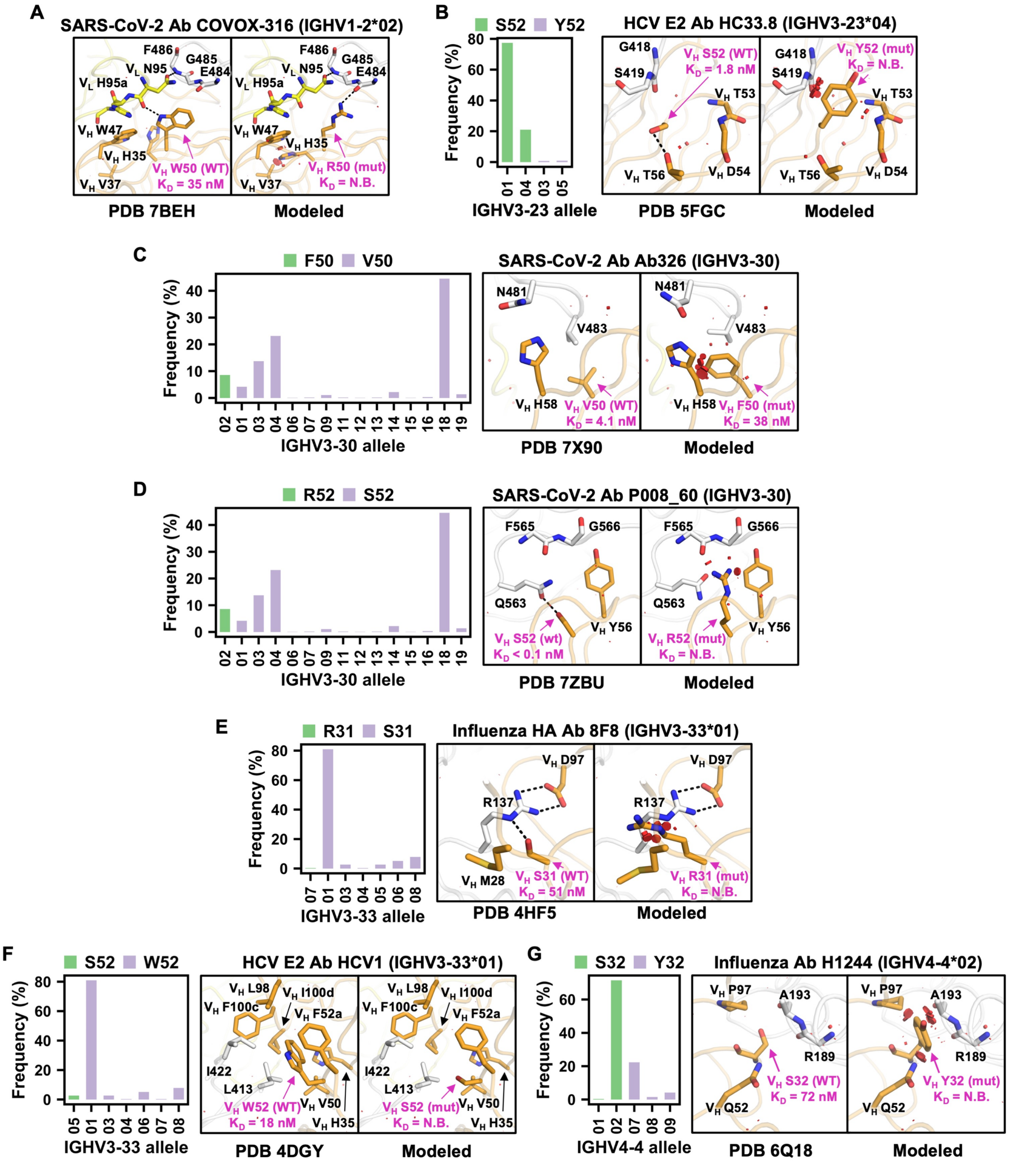

Figure S4. Structural analysis of selected paratope allelic mutations. The structural effects of paratope allelic mutations **(A)** V_H W50R of antibody COVOX-316 in complex with the receptor binding domain (RBD) of SARS-CoV-2 spike (PDB 7BEH) [1], **(B)** V_H S52Y of antibody H33.8 in complex with a peptide fragment of HCV E2 (PDB 5FGC) [2], **(C)** V_H V50F of antibody Ab326 in

complex with the RBD of SARS-CoV-2 spike (PDB 7X90) [3], **(D)** V_H S52R of antibody P008_60 in complex with SARS-CoV-2 spike monomer (PDB 7ZBU) [4], **(E)** V_H S31R of antibody 8F8 in complex with influenza H2N2 A/Japan/305/1957 hemagglutinin (PDB 4HF5) [5], **(F)** V_H W52S of antibody HCV1 in complex with a peptide fragment of HCV E2 (PDB 4DGY) [6], and **(G)** V_H S32Y of antibody H1244 in complex with influenza H1N1 A/Beijing/262/1995 hemagglutinin head domain (PDB 6Q18) [7], are modeled by FoldX [8]. The IGV gene and allele usage for each antibody are indicated. Of note, the allele usages for Ab326 and P008_60 cannot be assigned unambiguously. Binding dissociation constant (K_D) values of wild type and allelic mutant to the antigens were measured by BLI and are indicated. Structure visualization was generated using the same style and format as **Figure 4**. The bar charts on the right in each panel shows the allele usages of different IGV genes. Bar color represents the amino-acid identity at the indicated residue position.

Table S2. Summary of experimentally validated paratope allelic mutations.

PDB	Antibody	Antigen	Allelic mutation	WT freq (%)[a]	Predicted $\Delta\Delta G_{binding}$ (kcal/mol)[b]	WT K_D (nM)	Mutant K_D (nM)
8DXT	GAR12	SARS-CoV-2 spike	IGKV1-5 D50K	16.3	6.3	3.5	153
7BEH	COVOX-316	SARS-CoV-2 spike	IGHV1-2 W50R	91.8	17.2	35	No binding
7X90	Ab326	SARS-CoV-2 spike	IGHV3-30 V50F	91.5	5.7	4.1	38
8D36	COV44-62	SARS-CoV-2 spike	IGHV1-2 R50W	8.2	2.6	26	No binding
7ZBU	P008_60	SARS-CoV-2 spike	IGHV3-30 S52R	91.5	11.7	<0.1	No binding
4Z0X	HC84.62.5D	HCV E2	IGHV1-69 G50R	83.6	14.2	0.9	5.3
5FGC	HC33.8	HCV E2	IGHV3-23 S52Y	98.4	20.6	1.8	No binding
4DGY	HCV1	HCV E2	IGHV3-33 W52S	97.3	2.4	18	No binding
4XBE	4E10	HIV Env	IGHV1-69 G50R	83.6	12.6	9.1	No binding
5GJS	3E1	Influenza HA	IGHV4-4 R50E	22.3	1.4	6.9	No binding
6Q18	H1244	Influenza HA	IGHV4-4 S32Y	72.0	22.2	72	No binding
4HF5	8F8	Influenza HA	IGHV3-33 S31R	99.7	17.9	51	No binding
4M5Z	5J8	Influenza HA	IGLV3-21 D50Y	66.3	2.3	4.0	No binding
7RXI	Fab234	*P. falciparum* CSP	IGLV3-21 D50Y	66.3	8.5	0.2	1.0

[a] Occurrence frequency of individual alleles of the indicated germline genes was estimated based on antibody sequences downloaded from GenBank (www.ncbi.nlm.nih.gov/genbank) [9] (**see Methods**). The occurrence frequency of alleles that encode the wild-type (WT) amino acid at the indicated position of the corresponding antibody is listed. For example, antibody GAR12 is encoded by IGKV1-5 with Asp50. Among all the IGKV1-5 antibodies in GenBank, 16.3% were assigned to IGKV1-5 alleles that encode Asp50 in the germline.

[b] $\Delta\Delta G_{binding}$ was predicted using FoldX [8].

Supplementary References

1. Dejnirattisai W, Zhou D, Ginn HM, Duyvesteyn HME, Supasa P, Case JB, et al. The antigenic anatomy of SARS-CoV-2 receptor binding domain. Cell. 2021;184(8):2183-200.e22. Epub 2021/03/24. doi: 10.1016/j.cell.2021.02.032. PubMed PMID: 33756110; PubMed Central PMCID: PMCPMC7891125.

2. Keck ZY, Girard-Blanc C, Wang W, Lau P, Zuiani A, Rey FA, et al. Antibody response to hypervariable region 1 interferes with broadly neutralizing antibodies to hepatitis C virus. J Virol. 2016;90(6):3112-22. Epub 2016/01/08. doi: 10.1128/JVI.02458-15. PubMed PMID: 26739044; PubMed Central PMCID: PMCPMC4810650.

3. Takeshita M, Fukuyama H, Kamada K, Matsumoto T, Makino-Okamura C, Uchikubo-Kamo T, et al. Potent SARS-CoV-2 neutralizing antibodies with therapeutic effects in two animal models. iScience. 2022;25(12):105596. Epub 2022/11/22. doi: 10.1016/j.isci.2022.105596. PubMed PMID: 36406861; PubMed Central PMCID: PMCPMC9664764.

4. Seow J, Khan H, Rosa A, Calvaresi V, Graham C, Pickering S, et al. A neutralizing epitope on the SD1 domain of SARS-CoV-2 spike targeted following infection and vaccination. Cell Rep. 2022;40(8):111276. Epub 2022/08/19. doi: 10.1016/j.celrep.2022.111276. PubMed PMID: 35981534; PubMed Central PMCID: PMCPMC9365860.

5. Xu R, Krause JC, McBride R, Paulson JC, Crowe JE, Jr., Wilson IA. A recurring motif for antibody recognition of the receptor-binding site of influenza hemagglutinin. Nat Struct Mol Biol. 2013;20(3):363-70. doi: 10.1038/nsmb.2500. PubMed PMID: 23396351; PubMed Central PMCID: PMCPMC3594569.

6. Kong L, Giang E, Robbins JB, Stanfield RL, Burton DR, Wilson IA, et al. Structural basis of hepatitis C virus neutralization by broadly neutralizing antibody HCV1. Proc Natl Acad Sci U S A. 2012;109(24):9499-504. Epub 2012/05/25. doi: 10.1073/pnas.1202924109. PubMed PMID: 22623528; PubMed Central PMCID: PMCPMC3386053.

7. McCarthy KR, Raymond DD, Do KT, Schmidt AG, Harrison SC. Affinity maturation in a human humoral response to influenza hemagglutinin. Proc Natl Acad Sci U S A. 2019;116(52):26745-51. Epub 2019/12/18. doi: 10.1073/pnas.1915620116. PubMed PMID: 31843892; PubMed Central PMCID: PMCPMC6936356.

8. Delgado J, Radusky LG, Cianferoni D, Serrano L. FoldX 5.0: working with RNA, small molecules and a new graphical interface. Bioinformatics. 2019;35(20):4168-9. Epub 2019/03/16. doi: 10.1093/bioinformatics/btz184. PubMed PMID: 30874800; PubMed Central PMCID: PMCPMC6792092.

9. Benson DA, Cavanaugh M, Clark K, Karsch-Mizrachi I, Lipman DJ, Ostell J, et al. GenBank. Nucleic Acids Res. 2013;41(Database issue):D36-42. Epub 2012/11/30. doi: 10.1093/nar/gks1195. PubMed PMID: 23193287; PubMed Central PMCID: PMCPMC3531190.
